# Imported malaria cases in Sukabumi District-West Java Indonesia, in 2012

**DOI:** 10.1186/1475-2875-11-S1-P94

**Published:** 2012-10-15

**Authors:** Tris Eryando, Dewi Susanna, Dian Pratiwi, Fajar Nugraha

**Affiliations:** 1 Department Biostatistic and Health Informatics, Faculty of Public Health, Universitas Indonesia, Kampus Ul Depok, 16424, Indonesia; 2 Department of Environmental Health, Faculty of Public Health, Universitas Indonesia, Kampus Ul Depok, 16424, Indonesia; 3 Center For Biostatistics and Health Informatics, Faculty of Public Health, Universitas Indonesia, Kampus Ul Depok, 16424, Indonesia; 4 Center For Biostatistics and Health Informatics, Faculty of Public Health, Universitas Indonesia, Kampus Ul Depok, 16424, Indonesia

## Background

Sukabumi District is located in the southern area of West Java province. In 2004, a malaria outbreak occurred, 785 cases were reported and 8 of them died. During the last 3 years, the incidence of malaria has been constantly high. In 2009, 290 cases were reported while in 2010 and 2011, there were 316 and 273 cases reported. The malaria cases occurred in 14 sub districts out of 47 sub districts in Sukabumi District area[1]. The malaria endemic in Sukabumi District indicates a very low impact of the malaria elimination program in the district. Therefore it is a necessity to identify the characteristics and transmission of malaria in Sukabumi District as basis for the future malaria elimination program.

## Materials and methods

The research was conducted in 4 sub district out of 14 malaria endemic sub district in Sukabumi District West Java. Using a cross-sectional study, interviews were carried out for all malaria incidences that were reported during the period of January 2011 - April 2012 from Health Centers in 4 sub-districts, consists of 17 villages with stratification of MCI to HCI with API 1-<5%o. The total respondents were 204 people, which were visited at home.

## Results

The malaria cases in 4 subdistricts in Sukabumi were mostly import cases (71%) not indigenous cases. The respondents were mostly infected from the areas outside of Java Island. They were sent back home when they were found to be ill and they got treated in the Health Centre located in their homeland. The majority of the cases were people who worked in Sumatra Island (88.3%), Sulawesi (5.5%), Nusa Tenggara (3.4%) and Papua (2.8%). Most of the respondents were male (95%), in the productive age or 15-54 years old (93%). They worked in the mining sector, mostly working in night shifts (69%). After recovering from malaria, around 64.2% of the respondents return to their previous work location, where they got malaria. The types of Plasmodium found in the study area were *Plasmodium vivax* (88.2%), *P. falciparum* (7.4%) and Mix (4.4%). As much of 80.9% of the respondents received an ACT (Artemisinin Combination Therapy), due to the resistance of chloroquin and SP (sulfadoksin-Primethamin), which follows the rules of Ministry of Health for malaria elimination programme[2]. Due to the strategy of malaria eradication strategy [3,4] to achieve low transmission and substantial reductions in mortality and morbidity from malaria,[5] it is necessary for the people who travel to malaria-endemic areas and eventually settle in those areas to take anti-malaria drugs,[6] and avoid mosquito bites at night by using mosquito nets and or repellent.

## Conclusion

The most malaria cases in Sukabumi were imported malaria cases from outside of Sukabumi. They were in productive age male migrants who worked in mining sectors outside Java Island. Iit is necessary to educate, control environment, empower the community and to coordinate multi-sector in preventing malaria, by not letting the malaria cases transfered to another area or their homeland but to be treated in the endemic area.
